# A Plea for the Need to Investigate the Health Effects of Gig-Economy

**DOI:** 10.3389/fpubh.2021.638767

**Published:** 2021-02-09

**Authors:** Anna Freni-Sterrantino, Vincenzo Salerno

**Affiliations:** Department of Epidemiology and Biostatistics, Medical Research Council Centre for Environment and Health, Imperial College London, London, United Kingdom

**Keywords:** gig-economy, biomarkers, unstable jobs, stress, biological age

## Introduction

The gig economy is a rising phenomenon globally, where gig workers present “alternative work arrangements” (1) for pieces of jobs (“gigs”) or more generally short-term contract, which are mainly agreed upon via digital platforms (2) for different services, including food delivery or transportation. The gig job is a platform-based evolution of the “piece paid” job of the “80's, likewise transferring employers” economic risk-taking and responsibilities to individuals without a real reciprocal potential for gains in the form of increased pay or job security. Although there are generally empowering aspects of self-employment, including freedom of choosing gigs, the gig workers typically have little or no say on how much work is available. Under this kind of contracts, the work organization results in job instability, a risk of poor job quality and often low salaries (3). For these reasons, gig-workers represent a vulnerable population that is likely to be most exposed to stress.

Despite the difficulties in capturing these evolving work characteristics, there is evidence that the numbers of gig-workers are growing in U.S. and E.U. In 2016, Mc Kinsey (4) estimated that in U.S. 20–30% of the working-age population and the E.U. up to 162 million individuals, were engaged in some form of independent work, defined in the MC Kinsey report by: high degree of autonomy, payment by task, assignment, or sales and a short-term relationship between worker and client. In this generalization, the report included: “*free agents, who actively choose independent work and derive their primary income from it; casual earners, who use independent work for supplemental income and do so by choice; reluctants, who make their primary living from independent work but would prefer traditional jobs; and the financially strapped, who do supplemental independent work out of necessity.”* (4) In 2018, it was reported (5) that 36% of all the workers in the U.S. have alternative work arrangements and that by 2027, could increase up to 50% (6). In the U.K., between 2016 and 2019 numbers have doubled, while in Europe (7) 11% of the working population has performed some gig-work.

## Gig-Economy Studies State of Art

We have only a very partial picture of the health effects of the gig economy on workers, as data on gig jobs are fragmentary and research on health effects has only begun. However, some patterns and profiles are emerging. Gig work may be more prevalent in urban settings and among young people and immigrants at phases of entering the labor force. In the UK (8–10) a report conducted among 2,184 online respondents, indicated that the majority of gig workers are London based; half of them are 18–34 years old with educational attainment similar to that of the whole population. Fourteen percentage of gig workers have worked for more than 2 years and 38% between 6 months and 2 years in the gig market system. Overall, individual satisfaction is correlated with their employment status, i.e., negatively correlated when the gig-work is the primary source of income (9) and positively otherwise. Differences among types of gig workers and across countries have been reported (11) and governmental policies implemented to safeguard gig-workers' rights vary substantially. What is evident, from a public health perspective, is that the flexibility of such jobs goes hand-in-hand with *existential instability* (i.e., narrowing other domains of life, hampering partnering and starting families with potential for other adversities in individual adult life course), which is exacerbated among those who rely entirely on “gigs” for their income.

The gig economy may involve high work stress such as “job strain” or “effort-reward imbalance” as well as job insecurity that has been shown to have adverse effects on health (12–14). In a pooled analysis (*n* = 124,808) (15) job strain, the most widely studied form of work stress was associated with an increased risk for type 2 diabetes (16) in men and women independently of lifestyle factors. A meta-analysis (17) of published and unpublished results found that coronary risk disease was associated with job strain dimensions of job demand and job content, and with an attributable population risk for job strain of 3.4%. Job strain was found to be significantly increasing the risk of death in men with a cardiometabolic disease (18), independently of conventional clinical risk and lifestyle factors. The study authors concluded that “targeting conventional risk factors is therefore unlikely to mitigate the mortality risk associated with job strain in this population” (18). In the effort-reward imbalance model, stress is generated by the recurrent experience of a failed reciprocity between the effort spent at work and the rewards received in turn, material and non-material. Dragano et al. (19) have found, using a multicohort study of 90,164 employed individuals, that the effort-reward imbalance at work is associated with an increased risk of coronary disease. Mutambudzi et al. (20) found an association between effort-reward imbalance with an increased risk of diabetes incidence for subjects that worked 55 or more hours per week and had no insurance coverage than those working in blue-collar jobs. Both associations were independent of job strain. Job insecurity and job loss and discontinuous employment have also been found to affect health outcomes.

Research in the “1970 British Cohort Study” (21) confirms that those who have experienced a job loss while aged between 30 and 42 years showed increased risks of diabetes and hypertension, that worsened in the presence of debts. In the U.S. (22), women in “piece rate” jobs self-reported more frequent adverse health outcomes than salaried female workers. Changes in employment history for individuals who were unemployed, ejected or in precarious occupational positions led to a higher risk of developing poor health conditions (23), in particular during the economic crisis between 2007 and 2012. While it is known that financial crises affect the health status of those without a permanent contract, job stress impacts workers in permanent positions too. Bruner et al. (24) found a dose-response relationship between work stress and risk of obesity among civil servants, while no association was found between job strain and an increased risk of obesity (25). The picture that emerges is that job-related sources of stress like job demand, job content, effort-reward imbalance, insecurity, job loss, and unemployment contribute in different and possibly independent ways to well-being.

## The Role of Biomarkers

Recently, epidemiological studies have started to evaluate the role of biomarkers as both internal indicators (e.g., allostatic load) of the health status and predictors for adverse health outcomes.

According to WHO (26), a biomarker is “*any measurement reflecting an interaction between a biological system and a potential hazard, which may be chemical, physical, or biological. The measured response may be functional and physiological, biochemical at the cellular level, or a molecular interaction.”*

The allostatic load is defined “*as the cost of chronic exposure to fluctuating or heightened neural or neuroendocrine response resulting from repeated or chronic environmental challenge that an individual reacts to as being particularly stressful.”* (27) These indicators are useful because they allow to monitor and evaluate the health status before adverse health events occur. This characteristic makes them suitable in studies where the exposed population is relatively young, like gig workers.

Different types of biomarkers have been found associated with job characteristics, including work-related stress. In 2017, Siegrist and Li (28) summarized the literature findings, linking stressful work with a broad range of biomarkers. They found a robust association with heart rate variability, altered blood lipids, risk of metabolic syndrome, increased blood pressure, altered immune function, inflammation, and increased cortisol release. This study highlighted that altered biomarkers are involved in pathways associated with disadvantaged working conditions and stress-related conditions.

Economic insecurity has been investigated in the U.K. Household Longitudinal Study (29). Those who were consistently economically insecure (defined as a subjective measure of the perceived insecurity and inability to afford monthly expenses) had altered levels of high-density lipoprotein (HDL)-cholesterol, triglycerides, C reactive protein (CRP), fibrinogen and glycated hemoglobin, compared to the economically secure. The same dataset was used to compute the allostatic load, an indicator composed of 12 biomarkers representing multiple biological systems. The authors (30) compared the allostatic load for people who were unemployed with those recently re-employed. Results indicated that those who were re-employed and rated their job quality as poor (based on indicators including job anxiety, insecurity, dissatisfaction, and low pay) presented a higher allostatic load compared to the unemployment group. Different biomarkers have also been proven to play a role in the regulation of inflammatory and immune pathways, which, in turn, are associated with environmental stressors (31).

Besides clinical and blood-related biomarkers, newer epigenetic biomarkers, based on DNA methylation, have become available. Bakusic et al. (32) conducted a systematic review of human and animal studies on work stress, burnout and depression. They reported different methylation patterns of the brain-derived neurotrophic factor gene (*BDNF*) and increased global methylation in relation to aspects of mental health. This opens new avenues for estimating the role of gig economy in relation to epigenetic changes due to work stress factors.

Among epigenetic biomarkers, “epigenetic clocks” based on a combination of DNA methylation CpG sites rather than single-gene sites, have been shown to be reliable and promising indicators of biological age. “Age acceleration” assessed through DNA methylation has been found to predict all-cause mortality, frailty, several physical functions, psycho-social stress (31) and cancer (33, 34). Epigenetic clocks have been suggested to behave as an intermediate biological mechanism linking environmental exposures (including socioeconomic position) and late-life poor health outcomes and mortality. Fiorito et al. (35) found that the effects of low socioeconomic position are detectable through epigenetic clocks, which mediate the socioeconomic position effects on aging, starting early in life. However, the traditional measures of socioeconomic position need to be further developed in order to adequately distinguish the gig workers from other types of self-employed and employed individuals and to be able to compare and untangle the peculiar gig working characteristics and the induced health effects.

## Discussion

With governments supporting little or no measures for workers in the gig economy, the future of these workers looks uncertain and their health at risk. The recent COVID-19 pandemic has significantly impacted the economy of an estimated 70% of gig-workers (36) worsening their already precarious situation. The gig-worker population is still quite young, hence hard health outcomes are less likely to be expected in the short term, except under extreme circumstances. Likewise, the fragmented nature of these jobs, with workers rosters not easily accessible and the difficult to detect health effects, make epidemiological studies challenging. We believe that future research should concentrate on these two main paths:

1. Research on developing DNA-based biomarkers as stable and consistent indicators.
2. Research to understand how interventions using moderators (e.g., primary source of income, government policies) can improve working conditions that will promote long-term health benefits.

A starting point for these investigations could be access to a national cohort that combines detailed job and employment history and health data assessment, including DNA assays. For example, in the United Kingdom, the U.K. Household Longitudinal Study and in Finland, the Northern Finland Birth Cohort (NFBC) in 1966 and 1986 collect work data and biological samples.

In conclusion, we suggest that focusing on the health effects among gig workers is of great public health relevance and that biomarker studies represent an important and viable approach to conducting epidemiological investigations of health outcomes in this young and highly unstable population. Such epidemiological information could better inform policies to design and implement preventive health measures.

## Author Contributions

AF-S drafted the paper. VS conducted the bibliography research. Both authors contributed to the final version.

## Conflict of Interest

The authors declare that the research was conducted in the absence of any commercial or financial relationships that could be construed as a potential conflict of interest.

## References

[1] Spreitzer GM Cameron L Garrett L Alternative work arrangements: two images of the new world of work. Annu Rev Organ Psychol Organ Behav. (2017) 4:473–99. 10.1146/annurev-orgpsych-032516-113332

[2] Keith MG Harms PD Long AC Worker health and well-being in the gig economy: a proposed framework and research agenda. Entrepre Small Business Stress Exp Stress Well Being. (2020) 18:1–33. 10.1108/S1479-355520200000018002

[3] Wood AJ Graham M Lehdonvirta V Hjorth I. Good gig, bad gig: autonomy and algorithmic control in the global gig economy. Work, Employ Soc. (2019) 33:56–75. 10.1177/0950017018785616 30886460 PMC6380453

[4] Manyika J Lund S Bughin J Robinson K Mischke J Mahajan D Independent work: choice, necessity, and the gig economy. McKinsey Glob Inst. (2016) 2016:1–16.

[5] Perspective GS The Gig Economy and Alternative Work Arrangements (2018). Available online at: http://acrip.co/contenidos-acrip/gallup/2020/mayo/gallup-perspective-gig-economy-perspective-paper.pdf

[6] Union UaF 5TH Annual Report Freelancing in America 2018. (2018). Available online at: https://www.upwork.com/i/freelancing-in-america/2017/ (accessed June 2, 2020).

[7] Commission E Developments and Forecasts of Changing Nature of Work. (2020). Available online at: https://ec.europa.eu/knowledge4policy/foresight/topic/changing-nature-work/developments-forecasts-changing-nature-work_en (accessed June 2, 2020).

[8] Department for Business, Energy and Industrial Strategy The Characteristics of Those in the Gig Economy. London: Department for Business, Energy and Industrial Strategy (2018). Available online at: https://assets.publishing.service.gov.uk/government/uploads/system/uploads/attachment_data/file/687553/The_characteristics_of_those_in_the_gig_economy.pdf

[9] Department for Business, Energy and Industrial Strategy The Experiences of Individuals in the Gig Economy. London: Department for Business, Energy and Industrial Strategy (2018). Available online at: https://assets.publishing.service.gov.uk/government/uploads/system/uploads/attachment_data/file/679987/171107_The_experiences_of_those_in_the_gig_economy.pdf

[10] Taylor M Marsh G Nicol D Broadbent P Good Work: The Taylor Review of Modern Working Practices. London: Department for Business, Energy and Industrial Strategy (2017).

[11] Bajwa U Gastaldo D Di Ruggiero E Knorr L. The health of workers in the global gig economy. Glob Health. (2018) 14:124. 10.1186/s12992-018-0444-8 30563539 PMC6299501

[12] Bartley M Job Insecurity and Its Effect on Health. London: BMJ Publishing Group Ltd (2005). 10.1136/jech.2004.032235

[13] Virtanen M Nyberg ST Batty GD Jokela M Heikkilä k Fransson EI. Perceived job insecurity as a risk factor for incident coronary heart disease: systematic review and meta-analysis. Br Med J. (2013) 347:f4746. 10.1136/bmj.f4746 23929894 PMC3738256

[14] Ferrie JE Virtanen M Jokela M Madsen IEH Heikkilä K Alfredsson L. Job insecurity and risk of diabetes: a meta-analysis of individual participant data. Can Med Assoc J. (2016) 188:E447–55. 10.1503/cmaj.150942 27698195 PMC5135521

[15] Nyberg ST Fransson EI Heikkilä K Ahola K Alfredsson L Bjorner BJ. Job strain as a risk factor for type 2 diabetes: a pooled analysis of 124,808 men and women. Diabetes Care. (2014) 37:2268–75. 10.2337/dc13-2936 25061139 PMC4113178

[16] Mutambudzi M Javed Z. Job strain as a risk factor for incident diabetes mellitus in middle and older age US workers. J Gerontol B Psychol Sci Soc Sci. (2016) 71:1089–96. 10.1093/geronb/gbw091 27445405

[17] Kivimäki M Nyberg ST Batty GD Fransson EI Heikkilä K Alfredsson L. Job strain as a risk factor for coronary heart disease: a collaborative meta-analysis of individual participant data. Lancet. (2012) 380:1491–7. 10.1016/S0140-6736(12)60994-5 22981903 PMC3486012

[18] Kivimäki M Pentti J Ferrie JE David Batty G Nyberg ST Jokela M. Work stress and risk of death in men and women with and without cardiometabolic disease: a multicohort study. Lancet Diabetes Endocrinol. (2018) 6:705–13. 10.1016/S2213-8587(18)30140-2 29884468 PMC6105619

[19] Dragano N Siegrist J Nyberg ST Fransson EI Alfredsson L Bjorner JB. Effort–reward imbalance at work and incident coronary heart disease: a multicohort study of 90,164 individuals. Epidemiol. (2017) 28:619. 10.1097/EDE.0000000000000666 28570388 PMC5457838

[20] Mutambudzi M Siegrist J Meyer JD Li J. Association between effort-reward imbalance and self-reported diabetes mellitus in older US workers. J Psychosom Res. (2018) 104:61–4. 10.1016/j.jpsychores.2017.11.008 29275787

[21] Waynforth D. Unstable employment and health in middle age in the longitudinal 1970 British birth cohort study. Evol Med Public Health. (2018) 2018:92–9. 10.1093/emph/eoy009 29692897 PMC5906902

[22] Davis M Hoyt E. A longitudinal study of piece rate and health: evidence and implications for workers in the US gig economy. Public Health. (2020) 180:1–9. 10.1016/j.puhe.2019.10.021 31830705

[23] Sarti S Zella S. Changes in the labour market and health inequalities during the years of the recent economic downturn in Italy. Soc Sci Res. (2016) 57:116–32. 10.1016/j.ssresearch.2015.12.010 26973035

[24] Brunner EJ Chandola T Marmot MG. Prospective effect of job strain on general and central obesity in the Whitehall II Study. Am J Epidemiol. (2007) 165:828–37. 10.1093/aje/kwk058 17244635

[25] Kivimäki M Singh-Manoux A Nyberg S Jokela M Virtanen M. Job strain and risk of obesity: systematic review and meta-analysis of cohort studies. Int J Obes. (2015) 39:1597–600. 10.1038/ijo.2015.103 26041697 PMC4579559

[26] Organization WH International Programme on Chemical Safety (IPCS) Biomarkers and Risk Assessment: Concepts and Principles. Geneva: World Health Organization (1993) 57.

[27] McEwen BS Stellar E. Stress and the individual: mechanisms leading to disease. Arch Intern Med. (1993) 153:2093–101. 10.1001/archinte.1993.00410180039004 8379800

[28] Siegrist J Li J. Work stress and altered biomarkers: a synthesis of findings based on the effort–reward imbalance model. Int J Environ Res Public Health. (2017) 14:1373. 10.3390/ijerph14111373 29125555 PMC5708012

[29] Niedzwiedz CL Katikireddi SV Reeves A McKee M Stuckler D. Economic insecurity during the great recession and metabolic, inflammatory and liver function biomarkers: analysis of the UK household longitudinal study. J Epidemiol Community Health. (2017) 71:1005–13. 10.1136/jech-2017-209105-28855264 PMC5754862

[30] Chandola T Zhang N. Re-employment, job quality, health and allostatic load biomarkers: prospective evidence from the UK household longitudinal study. Int J Epidemiol. (2018) 47:47–57. 10.1093/ije/dyx150 29024973 PMC5837779

[31] Vineis P Avendano-Pabon M Barros H Bartley MJ Carmeli C Carra L. Special report: the biology of inequalities in health: the lifepath consortium. Front Public Health. (2020) 8:118. 10.3389/fpubh.2020.504530 32478023 PMC7235337

[32] Bakusic J Schaufeli W Claes S Godderis L. Stress, burnout and depression: a systematic review on DNA methylation mechanisms. J Psychosom Res. (2017) 92:34–44. 10.1016/j.jpsychores.2016.11.005 27998510

[33] Hannum G Guinney J Zhao L Zhang L Hughes G Sadda S. Genome-wide methylation profiles reveal quantitative views of human aging rates. Mol Cell. (2013) 49:359–67. 10.1016/j.molcel.2012.10.016 23177740 PMC3780611

[34] Horvath S. DNA methylation age of human tissues and cell types. Genome Biol. (2013) 14:3156. 10.1186/gb-2013-14-10-r115 24138928 PMC4015143

[35] Fiorito G McCrory C Robinson O Carmeli C Rosales CO Zhang Y. Socioeconomic position, lifestyle habits and biomarkers of epigenetic aging: a multi-cohort analysis. Aging. (2019) 11:2045. 10.18632/aging.101900 31009935 PMC6503871

[36] Forum WE Gig Workers Among the Hardest Hit By Coronavirus Pandemic. (2020). Available online at: https://www.weforum.org/agenda/2020/04/gig-workers-hardest-hit-coronavirus-pandemic/ (accessed June 2, 2020).

